# Intemperance of Old

**Published:** 1859-10

**Authors:** 


### INTEMPERANCE OF OLD.

A correspondent sends us the following, as copied verbatim from an old book, bearing date of sixteen hundred and ninety-three, (A. D. 1693.)

" Often consider with thyself, what a dangerous sin the sin of intemperance is. It is an inlet to all sin, and for that reason perhaps is not particularly forbidden in any of the commandments, because it is contrary to them all. Drunkenness may be called a breach of every one of the commandments, because it disposes men to break them all. What sin is it that a drunken man stands not ready to commit? Fornication, murder, adultery, incest, what not? And how doth this sin transform a man into a beast; and make him the shame, and reproach of human nature? Of the two, it is much worse to be like a beast, than to be a beast. The beast is what God has made it, but the drunkard is what sin and the devil has made him. Add to this that the intemperate man is his own tormentor, yea, his own destroyer; as appears by the many diseases and untimely deaths which surfeiting and drunkenness daily bring upon men. For as temperance and sobriety is the nurse and preserver of life and health, so excess is the occasion of self-murder; it is like the lingering poison, which though it works slowly, yet it destroys surely. Consider too, that intemperance is a sin which a man cannot presently repent of as soon as he has committed it. A drunken man is no more fit to repent, than a dead man; and what assurance hath any man, when drunkenness closes his eyes over night, that he shall ever open them again in this world? Consider how many have died in drunken fits, without being sensible of their condition, until they have been miserably surprised by the unconceivable torments of hell-fire.

